# Supplementary material for: The psychosocial dimensions of seborrheic dermatitis: A cross-sectional study on anxiety, personality, and quality of life
Source: PLoS One. 2025 Aug 26;20(8):e0331030. doi: 10.1371/journal.pone.0331030 (PMC12380286; doi:10.1371/journal.pone.0331030)
Supplement: Attachment — Submitted filename: Response to reviewers.docx [file pone.0331030.s003.docx]

**Response to Reviewers**

We thank the reviewers and the editor for their careful reading and valuable feedback, which helped us improve the quality and clarity of the manuscript. Below, we provide point-by-point responses to each comment

**Reviewer #1**

We sincerely thank the reviewer for their valuable insights. To address the concerns raised, we have taken the following steps:

**Comment 1:**
*The article has an interesting topic and various questionnaire tools have been used. Just one important point is that in many cases there are other intervening factors that can be effective in the relationship between variables. Various factors such as gender, age, race, lifestyle conditions and other mental and social diseases.*

**Response:**
We sincerely thank the reviewer for their valuable insights. In response:

1. **Demographic and environmental variables:** We have revised the Discussion section (paragraph 6) to explicitly acknowledge that gender and seasonal variation were weakly but positively correlated with DLQI scores, suggesting they may independently affect perceived quality of life.
2. **Confounding variables:** We now highlight that, although psychiatric comorbidities were excluded, other psychosocial and lifestyle factors may influence results and should be explored in future research.
3. **Study limitations:** These considerations have been added to the final paragraph of the Discussion as study limitations, and we recommend that future studies use multivariate models and broader sampling to further clarify these relationships.

**Reviewer #2**

**Comment 1:**
*“Presented manuscript looks like an extension of the paper ‘Depression, Anxiety Levels and Quality of Life in Patients with Seborrheic Dermatitis. Turk J Dermatol 2015;9(4):181–185.’ but with the use of alternative instruments and a higher number of involved patients.”*

**Response 1:**
We thank the reviewer for this observation and for bringing attention to the 2015 study by Baş et al. While there are thematic overlaps regarding anxiety and quality of life in seborrheic dermatitis (SD), our study introduces several important distinctions that contribute novel insights:

- **Psychological Focus:** We utilized the Beck Anxiety Inventory (BAI) rather than HADS, providing a more detailed and widely used tool for anxiety measurement in dermatology.
- **Personality Trait Analysis:** The inclusion of the Bortner Personality Scale allowed us to examine Type A/B personality characteristics, which have not been previously studied in SD.
- **Objective Clinical Severity:** We included SDASI (Seborrheic Dermatitis Area and Severity Index) to assess physician-rated disease severity and analyze its association with patient-reported outcomes—a novel addition compared to prior research.
- **Sample Size and Diversity:** Our sample size was more than double that of the 2015 study, improving the robustness of correlation analyses and enabling exploration of demographic and environmental modifiers (e.g., seasonal variation).
- **Conceptual Contribution:** A key contribution of our study is the analysis of discordance between objective severity (SDASI) and perceived burden (DLQI), which is central to developing multidimensional care strategies.

We have clarified these contributions in the revised **Introduction and Discussion** sections to emphasize the novelty and added value of our research.

**Comment 2:**
*“The title of the present manuscript (The Psychosocial Dimensions of Seborrheic Dermatitis: Implications for Multidimensional Disease Management) should be changed as there is nothing about management in it.”*

**Response 2:**
We appreciate the reviewer’s suggestion regarding the manuscript title. To better reflect the content and focus of the study, we have revised the title to:

**“The psychosocial dimensions of seborrheic dermatitis: A cross-sectional study on anxiety, personality, and quality of life”**

This revised title avoids implying clinical management content while maintaining a clear summary of the study’s psychosocial scope.

**Comment 3:**
*“Anxiety and QoL in patients with seborrheic dermatitis were well studied in multiple published articles and it is therefore especially important to underline what is new in the results of the presented manuscript.”*

**Response 3:**
We thank the reviewer for this insightful comment. We fully acknowledge that anxiety and quality of life (QoL) in seborrheic dermatitis (SD) have been addressed in previous research, frequently using instruments such as the Hospital Anxiety and Depression Scale (HADS) or the Beck Anxiety Inventory (BAI). While our study also utilized the BAI, it differs in several key aspects.

First, to our knowledge, this is the first study to apply the Bortner Personality Scale in an SD population, allowing us to explore how Type A/B behavioral patterns relate to perceived disease burden. Second, our comparatively larger sample size strengthens statistical power and generalizability.

Furthermore, we employed both physician-rated severity (SDASI) and patient-reported impact (DLQI) to examine the divergence between clinical and subjective disease burden—an approach that enables a more multidimensional understanding. Additionally, we analyzed the influence of demographic and contextual variables (such as gender and seasonal variation), which have often been overlooked or only briefly mentioned in prior work.

For example, Baş et al. (2015) reported elevated anxiety and reduced QoL in SD patients, but their study did not evaluate disease severity or personality dimensions. By contrast, our study extends the literature by incorporating intersecting clinical, psychological, and environmental factors, offering a more integrative perspective on the psychosocial burden of SD.

**Reviewer #3**

**Abstract**

**Comment 1:**
Include numerical data, such as correlation coefficients and p-values, in the abstract's results section.

**Response 1:**
Done. We have added key correlation coefficients and p-values to the Results section of the Abstract. For example: “SDASI showed a strong positive correlation with Physician Global Assessment (Rho = 0.815, p < 0.001)… DLQI moderately correlated with BAI and anxiety severity (Rho = 0.465 and Rho = 0.365, both p < 0.001)

**Comment 2:**
Recognize study limitations, such as cross-sectional design, lack of causation, and possible self-report bias.

**Response 2:**
A sentence recognizing the study’s limitations, including its cross-sectional design and reliance on self-reported instruments, has been added to the end of the Abstract.

**Comment 3:**
Consider clinical implications, such as routine anxiety screening in SD management.

**Response 3:**
We revised the abstract's final sentence to emphasize the potential utility of routine anxiety screening in SD management.

**Introduction**

**Comment 1:**
The introduction presents a comprehensive summary of seborrheic dermatitis… However, improvements are required in terms of language correctness, conciseness, and structural organization.

**Response 1:**

1. We removed excessive anatomical/biological detail and redundant phrases.
2. Repetitions such as reintroducing SD across paragraphs were eliminated.
3. The structure has been revised to improve clarity and focus on the psychosocial framework.

**Comment 2:**
The introduction contains instances of excessive detail. For example… "Seborrheic dermatitis (SD) is..." repeated across paragraphs.

**Response 2:**

The repetition of the term "seborrheic dermatitis (SD)" across consecutive paragraphs has been eliminated.

**Comment 3:**

Define “EPB” once, then use the abbreviation consistently. Ensure all terms like DLQI, SDASI, BAI are introduced properly if appearing.

**Response 3:**
The reference to “EPB” was removed during simplification of the Introduction, as it was not essential for the study’s psychosocial focus. We added the following clarifying sentence at the end of the Introduction:
*“This study aims to investigate the relationship between SD severity, anxiety, personality traits, and quality of life. By examining these psychosocial dimensions together—using the Seborrheic Dermatitis Area and Severity Index (SDASI), the Beck Anxiety Inventory (BAI), the Bortner Personality Scale, and the Dermatology Life Quality Index (DLQI)—the study provides a more integrated understanding of SD’s impact. Unlike previous research, it uniquely incorporates personality assessment via the Bortner Scale to better inform holistic, patient-centered management approaches.*

**Materials and Methods**

**Comment 1:**
Specify the categorical comparisons done using the "Pearson chi-square test," such as gender vs. severity. Explain why Spearman correlation (non-parametric) was chosen, such as "due to non-normal distribution of SDASI/DLQI scores**.**

**Response 1:**

1. We now specify where the Pearson chi-square test was applied (e.g., gender vs. SD severity).
2. We explained the use of Spearman correlations due to the non-normal distribution of the continuous variables.

These changes appear in the revised “Statistical Methods” section.

**Results**

**Comment 1:**
Incorporate essential findings from tables into the narrative rather than depending solely on tables. The writers cite "Table 1," "Table 2," and so on, but there is no concise synopsis of what each table contains other than a sentence.

**Response 1:**
The Results section has been rewritten to integrate findings directly from the tables into the text, including values and statistical significance.

**Comment 2:**
Effect sizes are not interpreted. For example: "Rho =.365" It would help readers interpret the phrase "a moderate correlation." Consider using parentheses to provide uniform interpretation (e.g., mild, moderate, strong).

**Response 2:**
Correlation values now include interpretation descriptors (e.g., “moderate positive correlation”).

**Comment 3:**

Divide the section into subheadings and paragraphs, like:

Demographics and Lifestyle Factors

Clinical Characteristics

Correlations Between Variables

Associations with SDASI and DLQI Severity

Anxiety and Personality Traits

**Response 3:**

The Results section has been restructured under thematic subheadings:

1. Demographics and Lifestyle Factors
2. Clinical Characteristics
3. Correlations Between Variables
4. Associations with SDASI and DLQI Severity
5. Anxiety and Personality Traits

**Comment 4:**
There were no significant differences for these variables regarding SDASI (p > 0.05). Which variables exactly? Repeat them or summarize.

**Response 4:**
The Results section now specifies all tested variables and clearly states when no statistically significant differences were found.

**Discussion**

**Comment 1:**
Divide the discussion into clear theme sections, such as:

Summary of key findings

Interpretation of psychosocial vs. clinical outcomes

Role of personality and anxiety

Seasonal and gender-related effects

Clinical implications

Limitations and future research

**Response 1:**
We thoroughly revised the Discussion to include the following improvements:

1. Organized into clear thematic subtopics (e.g., main findings, psychosocial-clinical contrast, personality/anxiety, demographic effects, limitations).
2. Emphasized the divergence between objective disease severity (SDASI) and subjective burden (DLQI).
3. Discussed personality traits in the context of chronic inflammatory disorders with added references [17–21].
4. Replaced causal language with association-based interpretations.
5. Limitations such as cross-sectional design, lack of control group, and self-reported instruments were clearly stated.
6. Compared results with previous studies, particularly Cömert et al. [14] and Baş et al. [17], to emphasize novelty.

**Comment 2:**

Soften causal wording. Change "stress as the major cause" to "stress was frequently reported as a flare trigger.

**Response 2:**

All causal language has been replaced with association-based phrasing.

**Comment 3:**

Some in-text references are not linked to citation numbers.

**Response 3:**

All references now include corresponding numbered citations in the reference list.

**Conclusion**

**Comment 1:**
The multidimensional method is valid, but a brief example (e.g., psychological screening + clinical assessment) would improve comprehension.

**Response 1:**
The final sentence of the Conclusion has been revised to include a concrete example:

“…such as combining BAI-based anxiety evaluation with SDASI assessment in routine visits.”
