# Supplementary material for: The psychosocial dimensions of seborrheic dermatitis: A cross-sectional study on anxiety, personality, and quality of life
Source: PLoS One. 2025 Aug 26;20(8):e0331030. doi: 10.1371/journal.pone.0331030 (PMC12380286; doi:10.1371/journal.pone.0331030)
Supplement: Attachment — Submitted filename: Reviewer Comment-01-08.docx [file pone.0331030.s004.docx]

We thank the Academic Editor and the reviewers for their thoughtful and constructive feedback on our manuscript titled *"The psychosocial dimensions of seborrheic dermatitis: A cross-sectional study on anxiety, personality, and quality of life"* (Manuscript ID: PONE-D-25-15883R1). We have carefully addressed all comments and revised the manuscript accordingly. In particular, we have:

- Rewritten the Conclusions in both the Abstract and the main text to ensure they are directly grounded in our study results and reflect the scientific novelty of our work;
- Integrated comparative data from previous studies, including control group findings, to contextualize our results with respect to the general population;
- Added a reference to a recent meta-analysis (Chen et al., 2025) to further support the interpretation of our findings;
- Revised the Discussion section to improve clarity, flow, and emphasis on our study's contributions;
- Updated the reference list and re-numbered citations as needed.

Below, we provide a point-by-point response to each reviewer comment, including detailed explanations of all modifications made in the manuscript. We hope that the revised version meets the journal’s standards for publication.

**Reviewer comment:**
Conclusions should be rewritten. Conclusions of the abstract in its present form may be written without any study. Conclusions should be based on study results. Finally, it is important to reflect the scientific novelty of the study in conclusions.

**Author response:**
We thank the reviewer for this valuable comment. In accordance with the recommendation, we have completely rewritten the Conclusion sections in both the main text and the Abstract to ensure that they are clearly grounded in the results of our study and reflect the scientific novelty of our work.

Specifically:

1. Generic statements were removed and replaced with data-driven conclusions (e.g., the lack of correlation between SDASI and anxiety, and the moderate correlation between DLQI and anxiety; Rho = 0.465, p < 0.001).
2. We emphasized the dissociation between clinician-rated disease severity and patient-reported burden.
3. We highlighted the novelty of the study, noting that it is among the few to concurrently evaluate clinical, psychological, and personality factors in SD patients.
4. The implications for clinical practice were also clearly stated, including the potential utility of routine psychological screening.

**The revised Conclusion (in manuscript) now reads as follows:**
*This study elucidates the multifactorial nature of seborrheic dermatitis, revealing that the condition is shaped by biological, psychological, and environmental influences. While clinical severity (SDASI) showed no significant associations with anxiety or personality traits, patient-perceived burden (DLQI) was moderately correlated with anxiety severity (Rho = 0.465, p < 0.001), highlighting the dissociation between physician-rated disease and patient experience. These findings underscore the importance of incorporating systematic psychosocial screening into SD management. Routine use of anxiety screening tools (e.g., BAI) alongside conventional clinical assessments may help identify patients in need of psychosocial support, improving patient-centered care. By concurrently evaluating clinical, psychological, and personality dimensions in a single SD cohort, this study contributes novel insights to the dermatological literature and reinforces the value of a multidimensional, biopsychosocial approach in managing chronic inflammatory skin conditions.*

**The Abstract results and conclusion was also rewritten as follows:**

**Results**

*SDASI demonstrated a strong positive correlation with Physician Global Assessment (Rho = 0.815, p < 0.001), confirming its validity as a clinical severity tool. DLQI exhibited moderate positive correlations with both BAI and anxiety severity (Rho = 0.465 and Rho = 0.365, p < 0.001), indicating that anxiety substantially contributes to patient-perceived disease burden. In contrast, SDASI showed no significant associations with anxiety levels or personality traits (all p > 0.05), highlighting a dissociation between physician-rated severity and patient-reported quality of life. Weak correlations were observed between DLQI and female gender (Rho = 0.159, p = 0.011) as well as seasonality (Rho = 0.145, p = 0.018).*

**Conclusion:** *Seborrheic dermatitis imposes a significant psychosocial burden that is more strongly linked to anxiety than to objective clinical severity. Our findings underscore the importance of integrating routine psychological screening—such as BAI assessment—into SD management to identify patients at risk of diminished quality of life. This study contributes novel evidence by concurrently evaluating clinical, psychological, and personality dimensions, reinforcing the need for a multidimensional, patient-centered approach to chronic inflammatory skin disorders.*

**Reviewer comment:**
Authors mentioned in the Discussion section elevated levels of anxiety in SD patients from two other studies but did not compare their own results with the general population. It can be especially important to support authors' conclusions.

**Author response:**
Thank you for this insightful comment. In response, we have revised the Discussion section to better contextualize our findings in relation to the general population. Specifically, we now reference the study by Cömert et al., which utilized a comparable methodology and reported an anxiety prevalence of 12.6% among healthy controls. In contrast, our cohort exhibited a markedly higher anxiety rate of 30.9%, reinforcing the notion that seborrheic dermatitis is associated with a disproportionate psychological burden.

Additionally, we incorporated the recent systematic review and meta-analysis by Chen et al. (2025), which reported a pooled anxiety prevalence of 19% and depressive symptoms in 21% of SD patients. This further strengthens the argument that the psychological impact of SD exceeds that observed in general or dermatological populations.

To improve overall clarity and cohesion, we also reorganized and edited several sentences in the Discussion. These changes help to better emphasize the novel aspects of our study—namely, the simultaneous evaluation of clinical severity, psychological symptoms, and personality traits.

We note that due to these additions, the reference list has been updated and re-numbered accordingly.

**Relevant revision in Discussion section:**
*Cömert et al., including a control group, found significantly higher anxiety prevalence in SD patients (32.5%) compared to healthy controls (12.6%), but no correlation with disease severity. Our results reflect this pattern, revealing no relationship between anxiety and SDASI, but a moderate correlation with DLQI, reinforcing the distinction between clinical observation and subjective burden. Further supporting these observations, a recent meta-analysis reported clinically significant anxiety in 19% of SD patients—comparable to atopic dermatitis (21%) and acne vulgaris (30%) (18). In our cohort, 30.9% of participants exhibited moderate-to-severe anxiety (BAI ≥16), exceeding the meta-analytic prevalence and closely paralleling the rate reported by Cömert et al. (14). Although our study did not include a control group, these comparisons underscore the substantial psychological burden associated with SD. By simultaneously evaluating clinical severity, psychological distress, and personality traits in a relatively large sample, our study contributes to a more comprehensive understanding of SD and reinforces the need for integrated psychosocial assessment in dermatological care.*
